# World AIDS Day 2020: Reflections on global and South African progress and continuing challenges

**DOI:** 10.4102/sajhivmed.v22i1.1205

**Published:** 2021-03-10

**Authors:** Yogan Pillay, Leigh Johnson

**Affiliations:** 1 Clinton Health Access Initiative, South Africa; 2 Centre for Infectious Disease Epidemiology and Research, School of Public Health and Family Medicine, University of Cape Town, Cape Town, South Africa

**Keywords:** HIV, prevention, treatment, viral suppression, PrEP

## Abstract

**Background:**

Reflecting on progress and challenges in meeting global human immunodeficiency virus (HIV) targets is often done ahead of World AIDS Day. This article reflects on progress and the continuing challenges in meeting targets in South Africa (SA).

**Objective:**

To review policy and implementation related progress and continuing challenges towards eliminating HIV as a public health threat by 2030.

**Method:**

Policy analysis and review of modeling data from Thembisa 4.3.

**Results:**

South Africa has made significant progress in the adoption of policies with two exceptions. While there are gaps in reaching the 90-90-90 implementation targets, progress has been made in the past decade.

**Conclusion:**

While progress has been made in the past decade towards the global targets, much work remains to ensure that HIV transmission is curtailed and those that require treatment are initiated on treatment and are virally suppressed.

## Introduction

South Africa (SA) recently commemorated World AIDS Day 2020. At the start of 2021, we reflect on areas ‘done well’ and those ‘yet to show progress’. Three recent documents provide independent data with which SA can achieve this and rededicate healthcare workers to eliminating human immunodeficiency virus (HIV) as a public health threat by 2030. These articles are, the World AIDS Day Report 2020: ‘Prevailing against pandemics by putting people at the centre’, published by the Joint United Nations Programme on HIV/AIDS (UNAIDS),^1^ the 2020 Global HIV Policy Report: Policy barriers to HIV progress^2^ and current South African estimates as described in the ‘Thembisa 4.3 model’, a report published by the University of Cape Town.^3^

The annual UNAIDS World AIDS Day Report^1^ focuses on progress in meeting targets and on areas of ongoing concern. Of the estimated 38 million people living with HIV (PLWH) globally, 12 million are not on treatment. In addition, in 2019, 1.7 million people were newly infected and 690 000 died of acquired immunodeficiency syndrome (AIDS)-related causes. Although the target was to have at least 30 million people worldwide on treatment by December 2020, actual numbers were 4 million off this goal! In September 2020, UNAIDS reported a shortfall in achieving its 90-90-90 global targets. By the end of 2019, these were only 81-67-59! Furthermore, the target of having ≥ 73% of all PLWH on antiretrovirals (ARVs) and exhibiting viral suppression by the end of 2020, is said to be ‘unlikely’.^4^ Even before the Coronavirus Disease 2019 (COVID-19) pandemic, the World Health Organization (WHO) had indicated that the global HIV response ‘was stalling’!^5^

**FIGURE 1:**
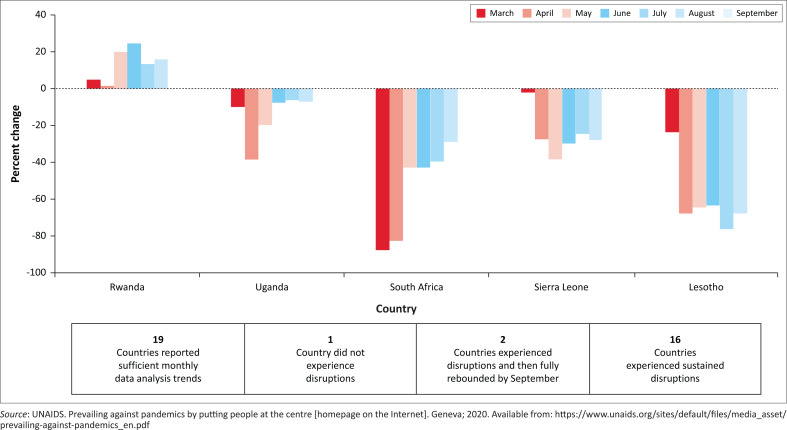
Change in the number of human immunodeficiency virus tests and results returned per month compared with baseline of selected countries in 2020.

Coronavirus Disease 2019 has negatively impacted the international HIV response. UNAIDS reports major disruptions in HIV-testing and access to antiretroviral therapy (ART).^1^ Access to HIV prevention services for men who have sex with men (MSM) was interrupted in Cambodia, Honduras, Jamaica, SA and Togo. In addition, the number initiating ART continued to decline through to September 2020, in the Dominican Republic, Kyrgyzstan, Lesotho, Sierra Leone and SA. Figure 1 indicates a rebound in testing in Uganda and SA between March/April and August/September 2020, but persistently poor testing rates in Sierra Leone and Lesotho.

The UNAIDS report^1^ also noted several positive initiatives prior to and in response to the COVID-19 pandemic. These include adopting new dispensing policies for stable HIV patients such as multi-month dispensing in Burundi, the Dominican Republic, Ethiopia, Mozambique, Papua New Guinea and SA. This reduced the need for clinic visits to collect ARV treatment. In Botswana, Kenya, Rwanda and SA, voluntary medical male circumcision (VMMC) has restarted since the imposition of strict lockdowns. Despite these initiatives, there is widespread concern about the disruption of essential services by COVID-19.

## South Africa’s policy related-progress and response to the HIV epidemic

Implementation of interventions to prevent HIV and get people onto treatment as soon as they are diagnosed starts with good policies. South Africa has long been feted as having exemplary policies. The 2020 Global HIV Policy Report^2^ shows that amongst countries in eastern and southern Africa – countries with the largest burden of HIV – SA has the highest overall policy-adoption score. The report suggests that SA has yet to make progress in the following three areas. The use of national laws to take advantage of the flexibilities in the Agreement on Trade-Related Aspects of Intellectual Property Rights (TRIPS) to procure medicines at more affordable prices, the decriminalisation of sex work and the personal possession and/or use of drugs. South Africa has used its large volumes and tender-pricing system to drive down national and global prices but has not utilised provisions of TRIPs such as compulsory licensing and parallel importation. Although the former Deputy President Mr. C. Ramaphosa publicly launched an HIV programme for sex workers and declared that ‘sex work *is* work’, this has not stopped police harassing sex workers.^6^ With respect to the legalisation of drug-use, to date only the private use of marijuana is legal but not its sale. However, drug use by the more than 75 000 injecting-drug users continues to be criminalised in SA, a situation that fuels both HIV and hepatitis transmission.^7^ South Africa must move more rapidly to a clearer policy on the decriminalisation of sex work and of injecting-drug use. In addition, the SA government must give greater consideration to the flexibilities of TRIPS where medicine prices are not being reduced to affordable levels.

## Changes in the HIV epidemic over the past decade: Estimates from the Thembisa model

In this report, we have used estimates from the Thembisa 4.3 model, an integrated HIV/demographic mathematical model developed for SA. This assesses the impact of different HIV programmes at a national and provincial level. Estimates from the Thembisa 4.3 model were released in November 2020 ahead of World AIDS Day 2020.

### Prevalence of HIV

The estimate is that there are 7.64 million people in the country living with HIV (PLWH: 4.84 million females ≥ 15 years, 2.49 million males ≥ 15 years and 310 000 children < 15 years). The total number of PLWH has increased from 5.9 million in 2010, an increase of 1.7 million between 2010 and 2019.

### Incidence of HIV

With respect to new HIV infections, there were 201 000 new HIV infections in 2018–2019: 121 000 in women, 67 000 in men and 11 600 infections from mother-to-child-transmission (MTCT). The majority of the latter (69%) occurred during the breastfeeding period. The total number of new infections in 2009–2010 was 412 000 – which implies a 51% decline in new infections over the 2010–2019 period. However, it is more meaningful to assess changes in HIV incidence rates in 15–49-year-olds. Changes in population growth and the population age distribution affect the absolute numbers of new infections. Using this metric, HIV incidence rates in SA declined by 55% over the 2010–2019 period, with the decline being greatest in KwaZulu-Natal (KZN) (61%) and least in the province of the Western Cape (34%). This decline in HIV incidence in KZN was confirmed in population-based surveillance studies.^8^ In terms of the drivers of these declines in KZN, high coverage of VMMC and greater access to ARVs have been suggested as contributory and recommended to be scaled up in other provinces.^9^

The highest Thembisa-model HIV incidence-rates are in MSM (2.60%) and female sex workers (FSWs) (5.50%). However, there have been significant declines in these cohorts. The incidence in MSM has declined from 5.69% and in FSWs from 10.96% in 2010. Although these are encouraging estimates, they largely reflect the impact of general HIV prevention and treatment programmes.^10^ Interventions such as pre-exposure prophylaxis (PrEP), which has been provided to FSWs and MSM, still have low uptake and retention rates. The model estimates a 2019 PrEP coverage rate of 3% and 1% in FSWs and MSM, respectively. These groups require special attention with respect to prevention and significantly increased access to treatment.

Over the past decade, there has been concern about HIV-infection in adolescent girls and young women (AGYW) aged 15–24 years. The Thembisa model estimates that the incidence rate in this cohort has declined from 2.98% in 2008 to 1.30% in 2018. These reductions are largely because of increased HIV testing, ARV coverage and high levels of condom use. Whilst still unacceptably high, this decline is important. Human immunodeficiency virus-incidence rates in adolescent boys and young men (ABYM) are lower in both 2008 and 2018 periods: 1.03% and 0.33%, respectively.^10^ Gender inequalities and transmission of HIV from older men to AGYW are posited as the main drivers of the incidence-differences between AGYW and ABYM.^11,12^

New infections at birth have declined from 18 300 in 2010 to 3600 in 2019: a decline of 80%. This is a consequence of the greater proportion of infected mothers on ART during pregnancy and at delivery, 32% in 2010 and 97% in 2019. However, the decline in new infections related to breastfeeding has been less dramatic: 22 000 (2010) to 8000 (2019) – a 63% decline. Breastfeeding mothers need greater support from healthcare professionals and their families/communities.

Pre-exposure prophylaxis in pregnant and breastfeeding mothers can reduce vertical transmission by 40%.^13^ Pre-exposure prophylaxis is safe in breastfeeding – with minimal infant-drug exposure.^14^ The use of PrEP as part of a comprehensive package of interventions in countries with a high HIV prevalence is endorsed by the WHO in both antenatal and postnatal care.^15^

### Prevention of HIV

Prevention starts with knowing one’s status. A combination of interventions accounts for prevention successes of the past decade. But greater effort is needed.

#### Test and treat

The Thembisa ‘ever-tested-for-HIV’ model estimates the percent of adults tested increased from 47.3% in 2010 to 76.3% in 2019. A total of 81% of SA women compared with 72% of men had ever-tested by 2019.

#### Condom use

Despite the provision of free male and female condoms by the SA government, their use at last sexual encounter increased marginally from 23% (2010) to 29% (2019) amongst women aged 25–49 years. Only 27% of 15- to 24-year-old females used a condom at their last sexual encounter. (Note that the Thembisa estimates are lower than self-reported rates as they are adjusted for social desirability-bias in self-reported data). As the most effective barrier method for the prevention of sexually transmitted infections (STIs) and unplanned pregnancies, more attention needs to be paid to convince South Africans of their value.

#### Circumcision

South Africa, supported by PEPFAR and the Global Fund, launched a large VMMC roll-out around 2008. The proportion of men aged 15–49 years who are circumcised has increased from 36.4% in 2010 to 57.5% in 2019.

#### Pre-exposure prophylaxis

The national SA-PrEP programme was introduced in 2016 and provided oral PrEP to sex workers. The programme was expanded in 2017 to include college and university students at onsite health clinics. Since 2018, PrEP has been provided in public health clinics.

### Deaths associated with HIV

The 2018–2019 estimate of HIV- and AIDS-related deaths in SA is 74 000 PLWH: 31 000 deaths in women, 39 000 in males and 3900 in children. The higher number of male deaths follows men presenting late to facilities, fewer men knowing their status, fewer men on treatment. Deaths have declined from 2009 to 2010 when it was estimated that there were 183 000 deaths – a 60% decline. As PLWH live longer, they are likely to need care for comorbidities such as diabetes, hypertension and cardiovascular diseases.^16^

### Antiretroviral coverage

Thembisa 4.3 estimates that ARV coverage increased from *n* = 530 877 (9.4%) in 2008 to *n* = 4 723 950 (62.7%) in 2018. Whilst this is a large increase in ARV coverage, males ≥ 15 years lag behind, increasing from 8.0% in 2008 to 57.2% by 2018. Women’s ART coverage was 9.9% in 2008 and 66.2% in 2018. Performing worst of all are children < 15 years of age: from 11.3% in 2008 to 53.2% in 2018.

Increasing access and improving adherence to ARVs for growing numbers of South Africans means accessing the latest, safest and best-tolerated ARVs at lowest cost. The newest (November 2019) of fixed-dose combinations in the SA public health sector is TLD: tenofovir (TDF), lamivudine (3TC) and dolutegravir (DTG). Whilst there are concerns about the long-term consequences of weight gain – greater in women than men, the combination is well tolerated and presents the virus with a high-barrier to resistance. Paediatric-DTG in a dispersible tablet is an important advance for infants and children living with HIV who are ≥ 4 weeks of age and weigh at least 3 kilograms (kg). Despite being endorsed by the WHO, paediatric-DTG has not as yet been registered for use in SA.^17^

### Meeting the UNAIDS 90-90-90 targets

How did SA do in the light of the UNAIDS’ 90-90-90 targets? The Thembisa estimates for adult women, males and children are presented separately to illustrate the variation across these groups (Table 1). Adult women in SA had reached 94-74-92 in 2019; adult males, 91-67-92 and children fared worst, 79-70-72. Table 1 indicates a great deal of provincial variation. KwaZulu-Natal is the best performing province: 95% of adult KZN-women know their HIV status, 77% are on ART and 95% of these are estimated to be virally suppressed. The worst performing provinces with respect to women living with HIV are Gauteng (92-69-88) and North West (92-64-91). The latter does ‘better’ with children at 82-70-70, compared with the worst performing province, Limpopo at 72-58-63.

**TABLE 1:** Progress towards the 90-90-90 targets in 2019.

Variable	Knowledge of HIV status (first 90% target)			Receiving ART if diagnosed (second 90% target)			Virally suppressed at < 1000 (third 90% target)		
	Adult male (%)	Adult female (%)	Children (%)	Adult male (%)	Adult female (%)	Children (%)	Adult male (%)	Adult female (%)	Children (%)
Eastern Cape	88.3	92.4	77.8	62.0	66.3	67.7	89.7	90.1	66.5
Free State	87.4	91.7	73.9	69.9	74.3	67.7	93.8	94.1	77.5
Gauteng	86.1	91.5	75.3	61.7	68.9	65.7	87.9	88.4	62.7
KwaZulu-Natal	92.0	94.9	79.1	74.8	76.7	68.2	94.5	94.7	78.7
Limpopo	89.5	93.3	71.6	63.5	70.1	58.0	88.1	88.7	62.9
Mpumalanga	88.9	92.9	72.5	68.4	72.3	64.7	91.7	92.0	71.1
Northern Cape	88.9	93.3	74.7	62.6	70.7	78.3	90.8	91.2	69.2
North West	86.7	91.8	81.8	54.1	63.7	70.3	91.0	91.4	70.2
Western Cape	88.5	93.1	77.2	62.9	68.4	72.6	93.6	94.0	77.1
National	90.6	94.2	78.9	66.6	73.6	69.8	91.8	92.3	72.2

*Source*: Johnson LF, Dorrington RE. Thembisa version 4.3: A model for evaluating the impact of HIV/AIDS in South Africa [homepage on the Internet]. 2020. Available from: https://www.thembisa.org/

HIV, human immunodeficiency virus; ART, antiretroviral therapy.

## Conclusion

At the beginning of each December since 1988, the global AIDS community and partners reflect on progress made in turning the tide on the HIV epidemic and consider the challenges as well as the work that remains to eliminate HIV as a public health threat. The year 2020 was especially challenging given the impact of the COVID-19 pandemic on individuals, families, and the local and global community. Health and social services were disrupted in many parts of the world making it difficult for people to access services. There is now a need for recovery and a reset to ensure that the gains made in the HIV programme, in SA and globally, are not lost and that we can accelerate towards the new targets that UNAIDS^18^ has proposed – of achieving the expanded 95-95-95 targets by 2025, as well as removing punitive laws, decreasing stigma and discrimination, and decreasing gender inequality and violence.
